# A novel RNA variant of human concentrative nucleoside transporter 1 (hCNT1) that is a potential cancer biomarker

**DOI:** 10.1186/s40164-019-0144-y

**Published:** 2019-08-22

**Authors:** Chunmei Wang, John K. Buolamwini

**Affiliations:** 1 Department of Pharmaceutical Science, College of Pharmacy, University of Tennessee Health Sciences Center, 881 Madison Avenue, Memphis, TN 38163 USA; 2 Department of Pharmaceutical Sciences, College of Pharmacy, Rosalind Franklin University of Medicine and Science, 3333 Green Bay Road, North Chicago, IL 60064 USA

**Keywords:** Concentrative nucleoside transporter 1 (SLC28A1), Splice variant, Intron retention, hCNT1 intron retention

## Abstract

**Background:**

The human concentrative nucleoside transporter 1 (hCNT1) a product of the *SLC28A1* gene is one of the three concentrative nucleoside transporters, with a substrate specificity for physiological pyrimidine nucleosides. It has recently been implicated in tumor suppression. We have unraveled a splice variant RNA transcript that is overexpressed in some tumor tissues and some cancer cells. This study established  that observation.

**Methods:**

We examined several clones of hCNT1 generated from RT-PCR of total RNA from human kidney tissue purchased from Ambion. The resulting cDNA clones were then sequenced, and a variant that retained intron 4, and skipped some exons fully or partly, specifically exons 5 and 13 were completely missed and only part of exon 6 was spliced. Tissue expression analysis by PCR indicated a similar distribution of expression of RNA of the splice variant hCNT1-IR as that of the dominant variant hCNT1, particularly in the small intestine, kidney and liver. Further, analysis of various tumor samples with PCR primers designed from this novel hCNT1 splice variant (hCNT1-IR) revealed interestingly that it is overexpressed in some cancer tissues relative to normal tissues, particularly kidney, liver and pancreatic cancers.

**Conclusion:**

We have identified a novel intron retaining and exon skipping splice variant of the hCNT1 nucleoside transporter, and designated it hCNT1-IR, which has a similar tissue expression distribution as the normal hCNT1 variant, but unlike the normal transcript, hCNT1-IR is overexpressed in some cancers and may serve as a potential cancer biomarker.

## Introduction

Physiological nucleosides and most therapeutic nucleoside analogues are hydrophilic molecules that require specialized nucleoside transporter (NT) proteins to pass across cellular membranes. NT-mediated transport is a critical determinant of intracellular nucleoside metabolism and the pharmacological actions of antineoplastic and antiviral nucleoside drugs [1, 2]. Nucleoside transporters are divided into two families: the SLC28 family comprising concentrative nucleoside transporters (CNTs) and the SLC29 family comprising equilibrative nucleoside transporters (ENTs). CNT1  is the first member of the CNT family that was cloned and functional characterized, and is predominantly expressed in kidney and jejunum [3]. CNT1 mediates the cellular uptake of naturally occurring pyrimidine nucleosides and adenosine to a much lesser extent, as well as diverse anticancer and antiviral nucleoside analogues including gemcitabine, cytarabine, and zidovudine [4–7].

CNT1 is a highly variable gene. A number of coding region single nucleotide polymorphisms (SNPs) have been reported for human CNT1 (see http://www.pharmgkb.org and http://www.pharmacogenetics.ucsf.edu). Sixty variable sites in the coding and flanking intronic regions of hCNT1 were identified in 247 DNA samples from an ethnically diverse population. Insertion (hCNT1 + Val) and deletion (hCNT1-1153del) mutations were also identified [8]. Initial cloning of human CNT1 from kidney yielded three highly similar clones, hCNT1a, hCNT1b, and hCNT1c, which may represent genetic variants of human CNT1 [3]. Coding region SNPs have also been reported for CNT1 by a study in a Japanese population [9]. The SNP database (dbSNP, http://www.ncbi.nlm.nih.gov/SNP) from that study lists the non-synonymous and synonymous changes for CNT1. In addition, one study has identified a cDNA variant of CNT1 with an additional 116 bp in the 5′-untranslated region from a human fetal liver cDNA library [10]. Gray et al. have studied the function of single amino acid variants of human CNT1 as a starting point in the analysis of genetic influence on variation in transporter function [11]. Variation in splice sites and promoter regions may also influence transporter expression or function. Hence, determining the functional roles of genetic and splice variants of concentrative nucleoside transporters in the targeting and disposition of naturally occurring nucleosides and synthetic nucleoside analogs is an important focus for future studies.

Alternative splicing is a very frequent phenomenon in the human transcriptome. There are four major types of alternative splicing: exon skipping, alternative 3′ splice site, alternative 5′ splice site, and intron retention [12]. Such variation can be functional or can represent mis-splicing. Alternative splicing can either produce functional alternative transcripts with distinct functions or might modulate functional spliced transcript level by producing some transcripts that do not encode proteins (sometimes by leaving some introns unspliced [13]. Intron retention, one form of alternative splicing, is common in plants but rare in higher eukaryotes, because messenger RNAs with retained introns are subject to cellular restriction at the level of cytoplasmic export and expression. In humans, 2–5 % of the genes have been reported to appear in the form of intron retentions [14, 15]. A recent study showed a splice variant of the organic cation transporter OCTN2 in which a 72-base-pair sequence located in the first intron of OCTN2 gene was spliced between exons 1 and 2, was retained in the endoplasmic reticulum, and did not transport carnitine [16]. Alternative pre-mRNA splicing increases proteomic diversity and provides a potential mechanism underlying both phenotypic diversity and susceptibility to genetic disorders in human populations. In the present study, we cloned a novel splice variant of hCNT1 with intron 4 retention from human kidney. The expression of protein and function of the variant were examined. We also studied the distribution of this hCNT1 intron retention transcript in several normal human tissues and some tumors, and observed variations in expression.

## Materials and methods

### Plasmid construction

RT-PCR was carried out with total RNA from human kidney (ClonTech) as a template using primers designated from the reported sequence of hCNT1a (GenBank accession number U62966): sense-EcoRV-hCNT1—5′-AGC *GAT ATC* TGG GAC ATG GAG AAC GAC-3′, antisense-SalI-hCNT1—5′-TTA *GTC GAC* TGT TCT GTC CTC ACT GTG CAC-3′ (restriction sites underlined). The PCR product was subcloned into FLAG tagged pCMV vector (Stratagene). Colony PCR was used to screen the positive colonies and a longer product was detected and sequenced (Molecular Resources Center, University of Tennessee Health Science Center, Memphis, TN).

### Cell culture

Porcine kidney tubular epithelium nucleoside transporter deficient cells (PK15NTD) were kindly donated by Dr. Chung-Ming Tse (Johns Hopkins University, Baltimore, MD). Human pancreatic cancer Panc-1, HPAC II and MIA PaCa-2 cell lines were purchased from ATCC (Manassas, VA). Cells were maintained in Eagle’s minimal essential medium/Earle’s Balanced Salt Solution with 0.1 mM nonessential amino acids, 1 mM sodium pyruvate (PK15NTD), Dolbeco’s minimum essential medium (Panc-1 and MIA PaCa-2) and 10% fetal bovine serum (Invitrogen, Grand Island, NY) and 2.5% horse serum additionally for MIA PaCa-2), Dulbecco’s modified Eagle’s medium and Ham’s F12 medium containing 1.2 g/l sodium bicarbonate, 2.5 mM l-glutamine, 15 mM HEPES and 0.5 mM sodium pyruvate supplemented with 0.002 mg/ml insulin, 0.005 mg/ml transferrin, 40 ng/ml hydrocortisone, 10 ng/ml epidermal growth factor and 5% fetal bovine serum (HPACII) at 37 °C in a humidified atmosphere of a mixture of 5% CO_2_ and 95% air.

### RNA extraction and RT-PCR

Total RNA of tumor cells (ATCC, Manassas, VA) was prepared using TRIzol reagent. 2 μg total RNA was treated with DNase I for 15 min to remove any genomic DNA that might be present, and subsequently used for cDNA synthesis. DNase-treated Human tissue total RNA was obtained from ClonTech, and cDNA was synthesized using SuperScript II Reverse Transcriptase (Invitrogen). PCR amplification was performed using the hCNT1 intron 4 primers. For tissue expression analysis, human tissue RNA arrays were purchased from Ambion for the above RT-PCR analyses.

The LightCycler system (Roche) was used for PCR under the following conditions: 95 °C for 5 min, followed by 40 cycles at 95 °C for 15 s, 60 °C for 30 s, and 72 °C for 10 s. The relative level of amplified RNA was normalized to mRNA expression of the housekeeping gene, glyceraldehyde-3-phosphate (GAPDH) or beta-actin (β-actin). Gene expression was evaluated by the ΔCT method using the GAPDH reference gene. All samples were amplified in duplicate and two non-template controls per primer pair were included in each run.

### Tissues

Human normal and tumor tissue total RNAs, i.e. normal kidney, kidney tumor, normal liver, liver tumor, normal pancreas, were purchased from Ambion and used to analyze hCNT1 and hCNT1-IR transcript expression by real time RT-PCR.

## Results

### A novel splice variant of hCNT1 was identified in normal human kidney

**Fig. 1:**
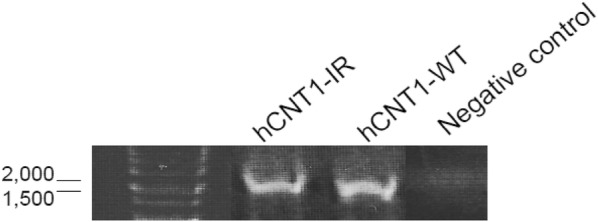
PCR colony products after cloning of hCNT1. A longer product (hCNT1-IR) was obtained after hCNT1 constructs were transform into *E. coli.* hCNT1-WT stands for the normal hCNT1 product

RT-PCR product of hCNT1 from normal human kidney total RNA obtained from a pool of 14 male/female Caucasians at ages between 18 and 58 years old was subcloned into a mammalian expression vector (pCMV-3flag). Colony PCR screening resulted in a longer product. Sequence alignment with full length hCNT1 cDNA showed that it contained an insertion of 734 bps from intron 4, between exons 3 and 4, and missing exons 5 and 13, as well as part of exon 6. We designated this novel splice variant of hCNT1 as hCNT1-IR (Fig. 1).

### hCNT1-IR is not translated

**Fig. 2:**
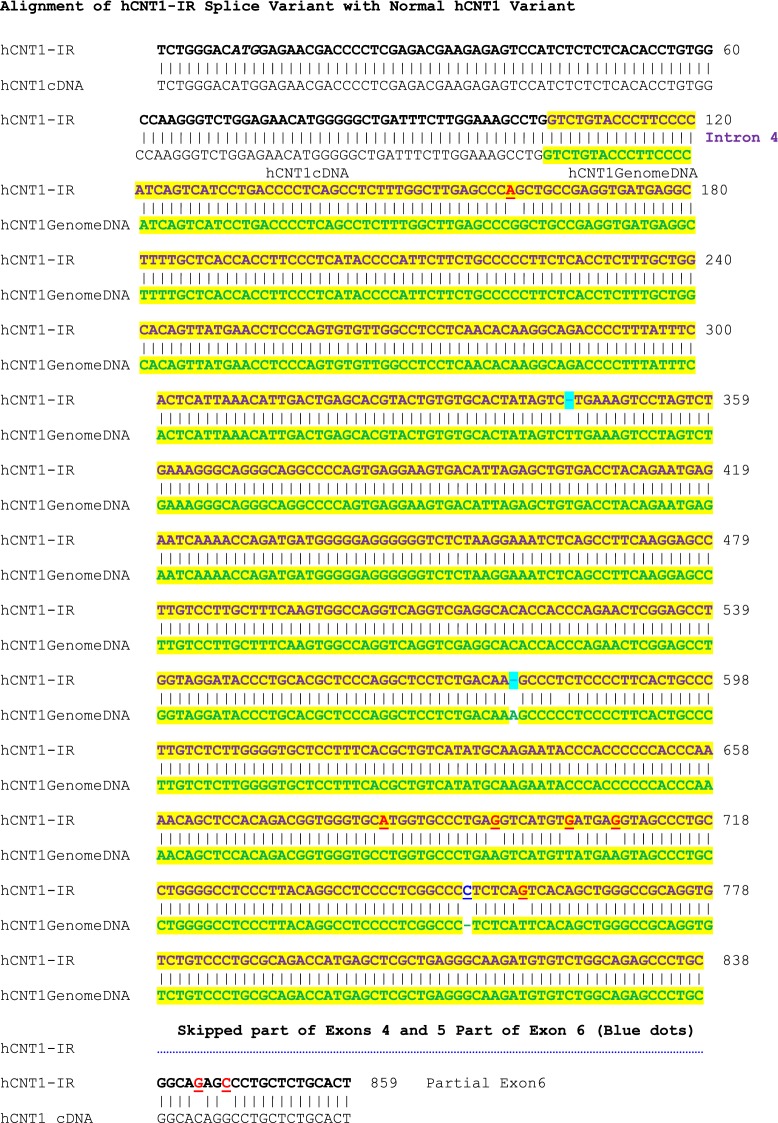

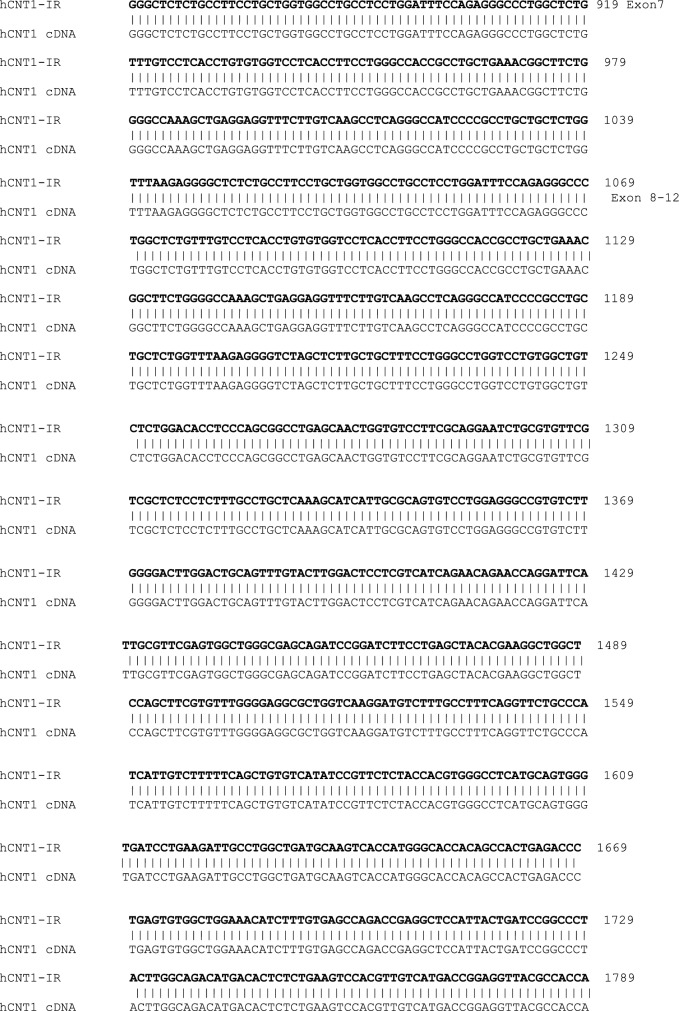

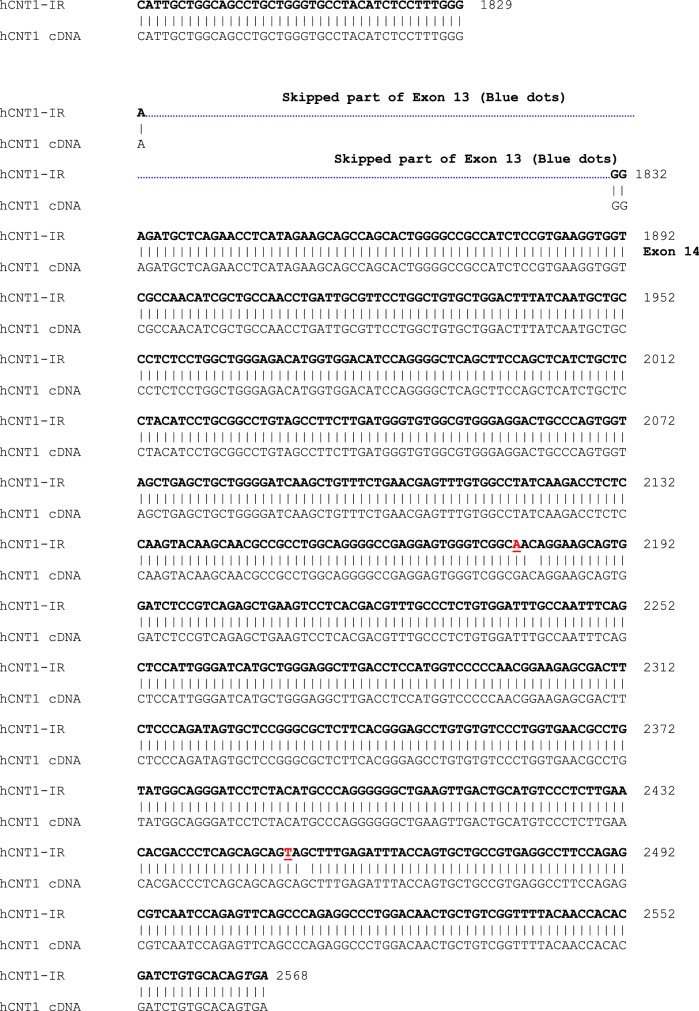
Sequence alignment of variant splice variant hCNT1-IR and normal hCNT1 transcripts. The top sequence is that of hCNT1-IR, while the bottom sequence corresponds to sequence of hCNT1. The initial and latter black bold face sequences are those of the initial and latter nucleotides of hCNT1-IR that are matched to corresponding to cDNA sequence of normal hCNT1 in black plain face nucleotides. In between these two regions and highlighted in yellow, are sequence of the retained intron 4 of hCNT1-IR in bold face purple, and the matching genomic sequence of normal hCNT1 in bold green sequence. Mutations in hCNT-IR sequence are indicated by red letters; insertions are indicated by blue letters, while deletions are indicated by cyan spaces. The skipped sequences are indicated by a blue dotted line

To determine whether the hCNT1-IR mRNA encodes protein, we transfected PK15 nucleoside deficient cells [17] with the hCNT1-IR construct, similar to what we have described previously [18]. Western blot analysis using anti-flag antibody according to that procedure [18] showed no protein expression; which could be the reason for no uridine uptake in a uridine transport assay [18] (data not shown). This is not surprising as the introduction of the intron and missing of exons caused a frame shift that probably precludes stable protein translation. The sequence of the hCNT1-IR splice variant showing retention of intron 4 and skipping of some exons is shown in Fig. 2. A BLAST analysis of this transcript sequence showed several stop codons and the largest possible reading frame only showed half the length of the normal hCNT1 cDNA open reading frame, which would be a very severely truncated protein that would probably be unstable even if translated.

### Tissue distribution and expression levels of hCNT1 intron 4 retention transcripts

**Fig. 3:**
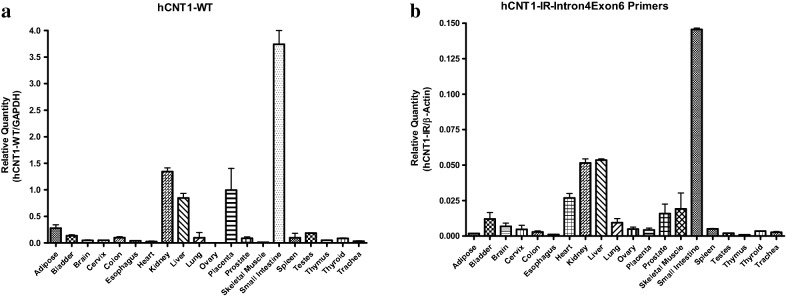
Tissue distribution of normal hCNT1-WT and intron retention variant transcript hCNT1-IR. For quantitative measurement of mRNA, DNase-treated human tissue total RNA human tissue total RNA panel was purchased from ClonTech. cDNA was synthesized using SuperScript II Reverse Transcriptase (Invitrogen). The PCR reactions using cDNA were performed in a LightCycler system (Roche) under the following conditions: 95 °C for 5 min, followed by 40 cycles at 95 °C for 15 s, 60 °C for 30 s, and 72 °C for 10 s. The relative level of amplified mRNA was normalized to mRNA expression of the housekeeping gene GAPDH (glyceraldehyde 3-phosphate dehydrogenase). Gene expression was evaluated by the ΔCT method relative to the reference gene. All samples were amplified in duplicate and two non-template controls per primer pair were included in each run. **a** qRT-PCR analysis of different human tissue RNA that using primers for normal hCNT1 transcript. **b** qRT-PCR analysis of different human tissue RNA using primers for intron retention hCNT1-IR splice variant transcript. Data are the mean ± SEM for three experiments run in duplicates

The tissue expression pattern of this splice variant transcript was quantified by real-time PCR expression studies for its levels in 20 different human tissues, using GAPDH as internal standard. The PCR results as shown in Fig. 3, showed that the intron retention variant hCNT1-1R (Fig. 3b) was most abundant in small intestine, followed by kidney and liver, and the heart, but was not high in the other tissues. As compared with the normal hCNT1 variant expression (shown in Fig. 3a), the expression pattern of hCNT1-IR is similar, except the overexpression of the latter hCNT1-IR in heart tissue (Fig. 3b). In the original cloning of CNT1 from the rat [19], a high stringency multi-tissue northern blotting with rat tissues showed predominant expression in jejunum (small intestine) and kidney tissues but no significant expression in other tissues, but a later study by using immunoblotting showed expression of rCNT1 in liver tissue as well, albeit at lower level than in small intestine and kidney [20]. The distribution of this novel hCNT1 intron retention variant, hCNT1-IR also shares similarities with that of rCNT1 except like with hCNT1, its significant overexpression expression in heart tissues (Fig. 3). The meaning of this is currently not apparent but may be worth studying.

### Overexpression of hCNT1-IR splice variant in some tumor tissues and cancer cell lines

**Fig. 4:**
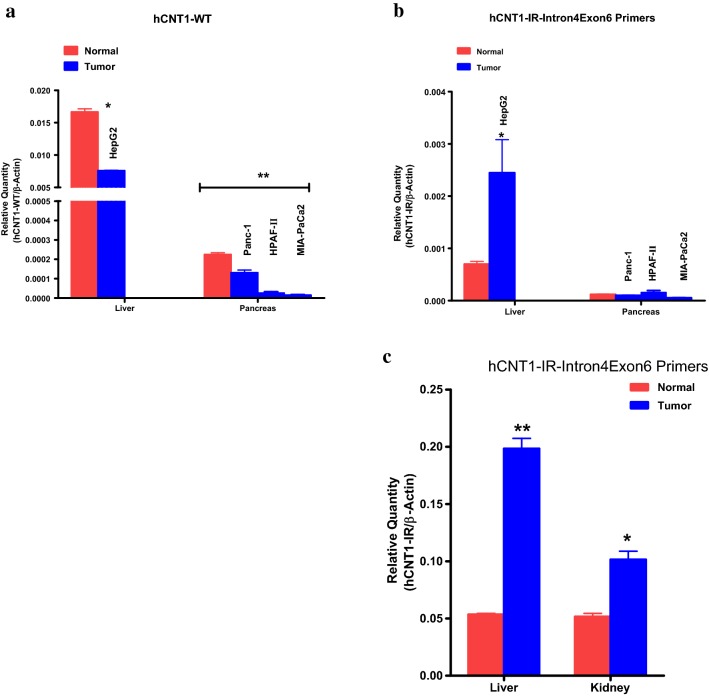
Comparative quantitative RNA analysis of hCNT1-IR or normal hCNT1 (hCNT-WT) transcript expression in human normal and tumor tissues or cancer cell lines. RNA was quantified from different tissues and cells using quantitative reverse transcription real-time-PCR (RT-qPCR). **a** Expression of normal hCNT1 transcript in normal human liver or normal human pancreas tissue (red), and in the human liver cancer cell line HepG2 or human pancreatic cancer cell lines Panc-1, HPAF-II and MIAPaCa2 (blue). **b** Relative expression of novel splice variant hCNT1-IR in the normal human liver and pancreas tissues (red) and in the human liver cancer cell line HepG2 and pancreatic cancer cell lines Panc-1, HPAF-II and MIAPaCa2 (blue). **c** Relative expression of novel splice variant hCNT1-IR in normal human liver or kidney tissue (red) and commercially available liver or kidney tumor tissue (blue). Each data point represents the mean and standard deviation of the mean from two independent experiments run in triplicate samples. *p < 0.05, **p < 0.01

We also looked at the differential expression of the normal hCNT1 and of hCNT1-IR transcripts. For the most part, the expression patterns of the two transcripts were similar, with small intestine expressing the highest levels, and substantial expression in the kidney and liver. However, in these normal tissues, there was some significant differences in expression also, being that while the kidney expressed more hCNT1 than the lever (Fig. 3a), the two tissues expressed the same level of hCNT1-IR (Fig. 3b). It is also notable that while hCNT1 expression level was substantial in the placenta, hCNT1-IR expression was not. Also, hCNT1-IR was shown to be expressed in heart tissue, while hCNT1 was not (Fig. 3). The significance of the similarities and differences in tissue expression patterns of the hCNT1 and hCNT1-IR variants is not apparent at this time. Important also is the distribution among the few tumor tissues that were examined. As shown in Fig. 4 interestingly, while normal tissues expressed higher levels of hCNT1 normal variant than tumor tissues, the opposite was true for the hCNT1-IR splice variant, which was overexpressed in tumor tissues relative to normal ones. In a study by Pennykooke et al. with human tumor tissues [21], hCNT1 was overexpressed in some kidney tumors, but there was generally a decrease in relative expression compared to normal kidney tissue. Our RT-PCR results of the expression of the intron retention variant in tumor samples shows an overexpression of hCNT1-IR in human kidney and liver tumor tissues, as well as significant overexpression in human hepatocellular carcinoma (HepG2) cell line compared to normal kidney and liver tissues (Fig. 4 a, b). The overexpression of hCNT1-IR in cancer relative to normal tissues was also demonstrated with pancreatic cancer cells whereby there was a significant downregulation of hCNT in cancer cells (Fig. 4a), but not in hCNT1-IR (Fig. 4b). A comprehensive analysis of hCNT1-IR expression in tumors and matched normal tissue is warranted.

## Discussion

The hCNT1 transporter protein is a widely expressed, high-affinity, pyrimidine-preferring, nucleoside transporter implicated in the uptake of physiological pyrimidine nucleosides as well as a variety of derivatives used in anticancer and antiviral treatment. hCNT1 is generally downregulated in cancer tissues and suspected of tumor suppressor activity. Here, we report a novel splice variant of hCNT1, hCNT1-intron retaining designated hCNT1-IR in human kidney and cancers. In hCNT1-IR, a 734-base pair sequence in the fourth intron of the hCNT1 gene was spliced between exons 3 and 4, while exons 5, 13 and part of exon 6 were skipped. hCNT1-IR protein was not detected in cells, and consequently no functional activity was observed. Interestingly, this intron-retention, exon skipping variant of hCNT1 was overexpressed not only in kidney but also in liver tumor, hepatocellular carcinoma and pancreatic cancer cells. This novel splice variant may be associated with pathological conditions of cancer, particularly kidney, liver and pancreas cancers, and cell lines, including HepG2, Panc-1, HPAC-II and MiaPaCa2 cells. Although the biological function of this variant remains to be elucidated, this is the first report of exon skipping and intron retention in nucleoside transporter RNA aberrant splicing. This study suggests that overexpression of this hCNT1 splice transcript may serve as a cancer biomarker.

Alternative splicing allows the production of multiple mRNA forms from one gene and therefore is crucial for protein diversity [22]. In addition, alternative splicing contributes to tissue and developmental stage-specific expression of protein isoforms. Besides yielding different proteins, alternative splicing in the 5′-noncoding region will not affect the gene product, but may influence gene expression and translation [22, 23]. Increasing evidence suggests that alterations in RNA processing can lead to a variety of human diseases, including cancer [22, 24]. It has been estimated that at least 15% of mutations that cause genetic diseases affect pre-mRNA splicing [25]. Indeed, numerous studies have indicated that alternative splicing occurs frequently in cancer cells, and a plethora of cancer-specific splice variants have been reported [26–28]; and some of them are considered to be candidate cancer biomarkers, such as *CD44* [28] and the Wilms tumor (*WT1*) genes [29]. The new hCNT1 splice variant did not show expression of protein, suggesting that the retained intron might restrict its translation or lead to frame shifts and/or stop codons that might prevent translation of stable protein(s). In this regard it has been reported that a frameshift deletion in the human equilibrative nucleoside transporter 3 (hENT3) coded by the *SLC29A3* gene led to a paradoxical translation of an otherwise noncoding mRNA splice variant [30]. The tissue expression pattern of the aberrant splice variant, hCNT1-IR is similar to that of the normal hCNT1. Intriguingly though, the aberrant splice variant is overexpressed in tumor tissue relative to normal tissues of the same organ origin as demonstrated by our analysis of normal and tumor tissues of kidney and liver, in particular. This is opposite to the general downregulation of hCNT1 in cancers. This suggests that the aberrant hCNT1 splicing may be overactive in disease conditions; at least in cancer. However, its physiological significance and possibly pathological function still need to be studied. Especially, it will be important to study the significance of hCNT1 aberrant splicing in the pathogenesis of tumors that express it, like kidney and liver cancers. Thus, as we propose, the hCNT1-IR splice variant might serve as a novel tumor biomarker, and possibly a therapeutic target. In broader terms, this study might open the door to studying such complex splicing of nucleoside transporter (NT) genes. Our study shows for the first time the combined intron retention and exon skipping splicing of any nucleoside transporter (NT) gene. A recent report described an insertion mutant of the hCNT3 (*SLC28A3*) gene that incorporated an extra 176 base pair nucleotide segment within exons 2 and 3 with the expressed a protein confined to the endoplasmic reticulum [31]. However, to date no intron retention and/or exon skipping nucleoside transporter transcript has been reported; and no variations in non-coding regions that might alter mRNA stability or transcription efficiency through polymorphisms in intron, or promoter regions or splice variation of NT genes have been described.

It is important to note that in sporadic amyotrophic lateral sclerosis (ALS) patients, multiple abnormal glutamate transporter EAAT2 (GLT-1) variant splicing with intron retention or exon skipping has been shown to result in the loss of EAAT2 protein activity but contributes to pathogenesis of the disease [32]. It is thus possible that in a similar manner, nucleoside transporter splicing such as we have unraveled in our study may contribute to pathogenesis of diseases linked to nucleoside homeostasis.

Our current study potentially opens the door to studying aberrant splicing in NT gene regulation, and in pathogenesis. It has been demonstrated that splice variant expression can be used as a means to regulate gene expression. An example is the EAAT2 transporter mentioned above, whereby abnormal splicing of the EAAT2 RNA repressed the normal EAAT2 protein expression [33]. This suppression of EAAT2 expression occurred to protect undifferentiated glioma cells from death due to the uptake of glutamate [32]. The rat, CNT1 gene expression is downregulated in liver tumors relative to normal tissue, with CNT1 protein being even absent in tumor lesions [34]. It has also been reported that hCNT1 is downregulated in human breast [35] and gynecological [36] cancers. In the gynecological cancer study, hCNT1 was by far the hCNT subtype that was most frequently downregulated or lost in the tumors analyzed. Moreover, hCNT1 protein loss was highly correlated with poor disease prognosis [35]. Conversely, the overexpression of hCNT1 has been shown to inhibit pancreatic cancer cell growth and increase sensitivity to chemotherapy [37]. The mechanism of downregulation of the usual hCNT1 variant in cancers noted above is unknown. As the aberrant splice variant expression of the EAAT2 glutamate transporter is used to suppress the normal variant expression in glioma cells [33, 38], the aberrant splice variant expression of hCNT1-IR may also be used to downregulate the expression on the normal hCNT1 variant in cancer.

## Conclusions

In conclusion, we have for the first time identified a novel splice variant transcript of hCNT1 that retains intron 4, skips exons 5 and 6, and part of exon 13. The tissue distribution of this new splice variant (hCNT1-IR) is similar to normal hCNT1 except in a few tissues like placenta which expresses hCNT1 and not hCNT1-IR, and heart tissue, which expresses hCNT1-IR but not normal hCNT1. Importantly, opposite to the pattern of normal hCNT1, which is usually downregulated in cancers, hCNT1-IR is overexpressed in some cancers relative to normal tissues. This could lay the foundation for developing a new cancer biomarker and/or cancer therapeutic target.

## Data Availability

The datasets collected and/or analyzed during the current study are available from the corresponding author on reasonable request.

## Abbreviations

CNTs: concentrative nucleoside transporters
ENTs: equilibrative nucleoside transporters
hCNT1: human concentrative nucleoside transporter-1
hCNT1-IR: human concentrative nucleoside transporter-1 intron retention
NT: nucleoside transporter
*SLC28A1*: solute carrier gene family 28 variant A1
SNPs: single nucleotide polymorphisms
